# Effects of forest park’s biological sounds on the positive responses of stressed university student

**DOI:** 10.1016/j.isci.2026.116073

**Published:** 2026-05-24

**Authors:** Tengfei Hui, Jie Zhang, Xuan Pei, Yang Zhi, Tong Song, Shiyu Song, Aiyun Zhu, Wei Gao, Chunlan Zhang

**Affiliations:** 1 Landscape Architecture Department, Forestry Institute, Beihua University, Jilin, China

**Keywords:** Human physiology, Behavioral neuroscience, Psychology

## Abstract

Given the widespread concern over university students’ psychological health and the significant role of biological sounds in psychological recovery, this study experimentally verified the effects of forest park biological sounds on stressed university students. We recruited 32 students (stress measured via the Perceived Stress Scale), used 11 biological sound samples from Henan forest parks as auditory stimuli, and tested their electroencephalogram (EEG) and psychological questionnaire in the laboratory. We find that biological sounds enhance security/relaxation and alleviate fatigue, with stronger effects in highly stressed students; *Oecanthus indicus* Saussure, *Hirundo rustic*, and *Ardea intermedia* exert the most positive effects, while *Corvus macrorhynchos* and *Tettigonia chinensis* Willemse have negative effects; *Hirundo rustica*, *Oecanthus indicus* Saussure, and *Passer ammodendri* score highest in psychological perception; and psychological and physiological responses correlate significantly. These findings highlight bioacoustics’ value in relieving student stress and provide data for future forest park landscape and soundscape design.

## Highlights

• Stressed students exhibit positive psychophysiological responses to biological sounds
• Higher stress levels in students lead to more positive responses to biological sounds
• EEG responses significantly correlate with psychological restoration scores
• Females and undergraduate students demonstrate higher levels of security/relaxation

## Graphical abstract

**Figure:**
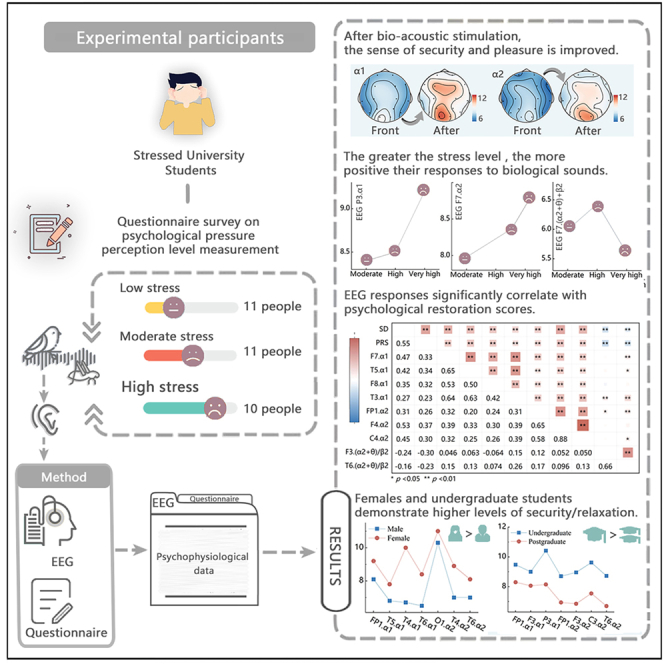

## Introduction

In today’s fiercely competitive society, students are confronted with various pressures from academics, social interactions, and employment,^1^^,^^2^^,^^3^ which significantly impact their physiological, psychological, and behavioral health.^4^^,^^5^^,^^6^ Research indicates that in most countries, more than half of university students have experienced varying levels of stress, anxiety, or depression.^7^ Garlow et al. reported that only 15% of the stressed university students with moderate to high depression or suicidal ideation in their sample were receiving treatment,^8^ and only a small number of stressed university students actively seek therapy when dealing with stress-related mental health issues.^9^

Exposure to forests has been widely proven to effectively improve mood, relieve stress and mental fatigue, and promote physical and mental recovery.^10^^,^^11^^,^^12^ Its benefits are not merely a matter of “natural preference” but are systematically explained by the stress recovery theory (SRT) and attention recovery theory (ART) in environmental psychology, which together constitute the core theoretical framework of the “restorative effect” of the natural environment. ART focuses on the cognitive aspect, believing that maintaining direct attention requires mental concentration and that long-term use can lead to fatigue, while the attractive natural environment can bring a sense of calm and comfort, helping to maintain and restore direct attention.^13^^,^^14^ Multiple studies have verified the recovery effect of natural exposure on stress and attention fatigue.^15^^,^^16^ SRT, on the other hand, focuses on the emotional and physiological responses triggered by natural environments. It indicates that exposure to nature can shift one’s attention from negative emotions to the natural landscape, replacing negative emotions with positive ones, restoring the balance of the physiological system, and thereby alleviating cognitive dysfunction caused by stress.^17^^,^^18^ This theory emphasizes the benefits of positive emotions. Relevant studies have also confirmed that observing natural environments can reduce stress, optimize physiological functions, improve cognition, and promote recovery.^19^^,^^20^ Together, these two aspects constitute the understanding of the restorative effect of natural environments. Biological sounds are an important component of the acoustic landscape in forest parks and a vital resource within the landscape.^21^ These sounds significantly influence people’s experience and play an essential role in psychological recovery.^22^^,^^23^ Therefore, studying the impact of biological sounds on stressed university students is highly important for the soundscape planning of parks and campuses. The earliest research on biological sounds was conducted in the areas of biological communication and function, revealing that biological sounds play an important role in territorial defense, attracting mates, deterring enemies, navigating, foraging, and maintaining the stability of social groups and ecosystems.^24^^,^^25^ In recent years, many scholars have researched the impact of biological sounds and found that they can eliminate unpleasant noise^26^ and have complex, variable, and plastic characteristics.^27^ From a health perspective, the perception of biological sounds can be used to improve the human experience,^28^ with bird songs being considered particularly restorative in urban parks^29^; help reduce mental illnesses such as depression, anxiety, and paranoia^30^; and integrate natural sounds (such as bird songs) in forest environments to enhance health benefits.^31^

Biological sounds have been demonstrated to exert significant dual effects on human psychological and physiological response systems. Initial research predominantly employed subjective methodologies, utilizing questionnaire surveys—often combined with sound walks and interviews—to investigate auditory preferences and perceived psychological benefits in natural settings.^32^^,^^33^ These foundational studies established that individuals exhibit a clear preference for biological sounds, particularly identifying bird songs as the most popular natural sound source for enhancing happiness^34^ and inducing feelings of calm.^35^ Bird calls were consistently linked to improved mental health, stress reduction, and decreased anxiety,^36^ while insect sounds were found to induce comfort and excitement, heighten immersion, alleviate mental burden,^37^ and facilitate faster recovery post-stress,^38^ collectively affirming biological sounds’ efficacy in boosting positive emotions and cognitive function while reducing stress. Demography is also an important factor influencing the perception of soundscapes. When dealing with stress and environmental stimuli, males and females exhibit different physiological response patterns and emotional expression tendencies.^39^^,^^40^ Furthermore, it has been found that an individual’s educational level is also closely related to their preference for soundscapes. Generally, individuals with higher educational attainment tend to prefer biological sounds and natural sounds more.^41^

Building upon these subjective findings, research progressively focused on quantifying physiological responses, utilizing objective metrics to verify stress reduction and restorative effects. Studies measuring skin conductance level (SCL) revealed that bird songs significantly lowered SCL compared to silence, water sounds, traffic noise, or light music.^42^ Broader investigation confirmed that sounds like bird songs and cicada calls consistently reduce physiological stress markers, including SCL, heart rate, and heart rate variability (HRV), and strengthen its pressure recovery function.^43^ Concurrently, physiological methods like eye-tracking—analyzing fixation rate, duration, saccade amplitude, and pupil diameter—demonstrated that bird and insect calls reduce cognitive load, enhancing immersion and lessening mental burden.^44^ Experiments with skin conductance, heart rate, and eye movement have demonstrated that biological sounds can cause physiological changes in humans. Studies have shown that auditory stimulation is highly correlated with neural response processes; consequently, studying the changes in brain waves before and after biological sound stimulation is essential to understand the physiological impact of biological sounds. Currently, electroencephalogram (EEG) is a widely used physiological measurement method in many disciplines, including medicine, psychology, and computer science.^45^

EEG, featuring millisecond-level temporal resolution, is especially well suited for monitoring the brain’s rapid neurophysiological responses to brief auditory stimuli, such as biological sounds.^46^ It has been recognized as a reliable tool of studying brain activity associated with emotions, attention, relaxation, and stress.^47^^,^^48^ Specifically, oscillations in the alpha frequency band are widely regarded as an electrophysiological marker for relaxation and calming the mind. The α1 waves are closely related to an individual’s sense of security, while the α2 waves are associated with an increase in overall relaxation levels.^49^ The ratio of (α+θ)/β is also used as a sensitive indicator of mental fatigue. A decrease in this ratio indicates a reduction in fatigue.^50^ Relevant studies have shown that after experiencing autumn-colored plants through virtual reality devices, significant changes in the α1 and α2 brain waves were observed in the control group and the experimental group in three different stimulation modes (visual only, auditory only, and combined visual and auditory). Among them, the EEG values of the auditory stimulation group showed the highest frequency of change.^51^ When examining the fatigue sensation caused by watching 3D TV, it was observed that (α+θ)/β showed a greater increase on a single electrode.^52^ These results prove that it is feasible to study the impact of sound on humans through physiological EEG measurement, providing a basis for using EEG to study the influence of biological sounds. Therefore, brain waves can be employed to objectively quantify the impact of the body on the brain under stress, thus complementing subjective psychological reports. College students, as a representative group of young people, exhibit stable brain wave activity, which facilitates the accurate recording and analysis of the influence of biological sounds on brain activity.

Despite the well-documented role of biological sounds in stress reduction, several critical gaps remain.^53^^,^^54^ Firstly, existing studies often focus on the general population, lacking targeted investigation into stressed university students as a specific vulnerable group. Secondly, while both psychological and physiological responses have been explored, research that deeply integrates subjective questionnaires with objective EEG measures to unravel the underlying psychophysiological mechanisms is still limited. Thirdly, many studies treat “biological sounds” as a homogeneous category, overlooking the potentially distinct effects of different sound sources and species, which hinders the development of precise, evidence-based soundscape design.

To address these gaps, this study specifically recruits university students with varying levels of perceived stress and employs a multi-method approach combining EEG measurements and psychological questionnaires under controlled laboratory conditions. We aim to experimentally verify the effects of 11 distinct biological sounds from forest parks in Central China on stressed university students. Our research hypotheses are as follows.

(1) Biological sound exposure will enhance security and relaxation and reduce fatigue relative to baseline.
(2) Bird songs will induce more positive psychological and physiological responses than insect sounds.
(3) Students with higher self-reported stress will benefit more from biological sounds, showing greater gains in security, relaxation, and fatigue reduction.
(4) Subjective psychological perceptions will be significantly positively correlated with the objective physiological responses and significantly negatively correlated with the physiological index of fatigue.

## Results

### Effects of different stressed university students

**Figure 1:**
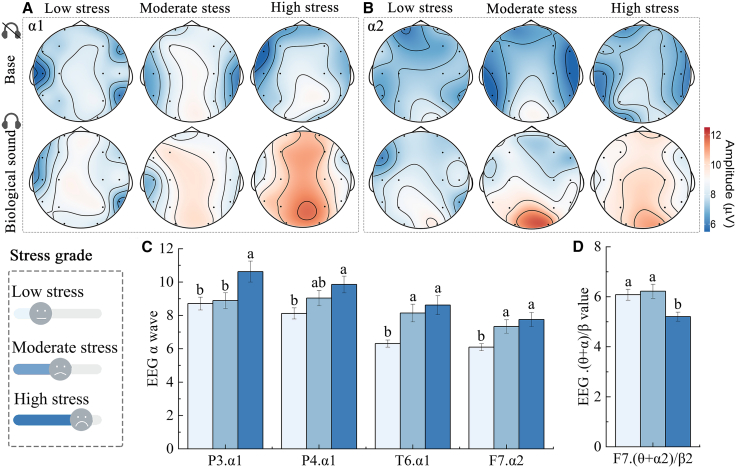
Comparison of EEG in university students with different stress levels under biological sound stimulation (A and B) Changes of EEG topographic map before and after bio-acoustic stimulation. (C and D) EEG values of university students with different stress levels after bio-acoustic stimulation. Data are presented as mean ± Standard deviation. Lowercase letters (a, b) indicate significance, and different letters indicate significant differences between groups. P3.α1, P3 channel α1 wave; P4.α1, P4 channel α1 wave; T6.α1, T6 channel α1 wave; F7.α2, F7 channel α2 wave; F7. (θ+α2)/β2, F7 channel (θ+α2)/β2 value.

As shown in Figures 1A and 1B, the EEG activity plots indicate that biological sounds can cause fluctuations in the brainwave energy of university students at different stress levels. Subsequently, we conducted *t* tests on university students at different stress levels to explore the physiological changes in the EEG before and after biological sound stimulation (Table 1). The results showed that the α1 and α2 waves of university students at different stress levels were significantly higher than the baseline level after the bio-acoustic stimulation, while the (θ+α)/β value decreased. This indicates that university students at different stress levels (including low stress, moderate stress, and high stress) would feel a stronger sense of security and pleasure in the biological acoustic environment, and their fatigue was also relieved to a certain extent. Among them, the effect of biological sounds on enhancing the sense of security and alleviating fatigue among university students under high stress is the most significant, followed by those under medium stress and finally those under low stress. In terms of enhancing a sense of relaxation, the biological sounds had the most significant impact on university students who were under moderate stress, followed by those under high stress and those under low stress (Table 2).

**Table 1:** Participant profile

Variable	Category	Number of people	Percentage
Stress grade	low	11	34.40%
Stress grade	moderate	11	34.40%
Stress grade	high	10	31.30%
Gender	male	15	46.90%
Gender	female	17	53.10%
Academic degree	undergraduate	16	50%
Academic degree	postgraduate	16	50%

**Table 2:** Comparison of EEG before and after biological sounds in university students with different stress levels

Pressure grade	Channels and EEG	Base	Biological sounds	Relative change value	*p*	*t*	df	Cohen’s *d*
Low stress	T3.α1	5.51 ± 0.40^b^	6.83 ± 0.29^a^	23.96% ↑	0.001	−2.66	23	1.11
Low stress	FP1.α2	5.90 ± 0.80^b^	7.71 ± 0.20^a^	30.68% ↑	0.001	−2.61	130	0.46
Low stress	T4. (θ+α2)/β1	4.00 ± 0.30^a^	3.43 ± 0.1^b^	−14.04% ↓	0.031	−2.27	130	0.50
Moderate stress	T4.α1	6.57 ± 0.60^b^	8.33 ± 0.56^a^	25.67% ↑	0.042	−2.14	33	0.75
Moderate stress	T4.α2	5.53 ± 0.45^b^	8.68 ± 0.48^a^	56.22% ↑	0.001	−4.79	42	1.40
Moderate stress	T6.α2	6.13 ± 0.56^b^	8.89 ± 0.47^a^	45.90% ↑	0.001	−3.78	28	5.02
Moderate stress	P4. (θ+α2)/β2	6.61 ± 0.17^a^	5.45 ± 0.20^b^	−18.18%↓	0.001	−4.52	28	1.77
High stress	F3.α1	7.29 ± 0.43b	10.48 ± 2.06a	43.76% ↑	0.038	2.04	118	4.76
High stress	F4.α2	6.61 ± 0.68b	11.12 ± 1.75a	53.10% ↑	0.048	1.83	9	3.32
High stress	P3. (θ+α1)/β2	8.48 ± 0.51a	6.57 ± 0.31b	−22.35% ↓	0.001	−3.21	77	5.12

a and b，Different lowercase letters (a, b) indicate significant differences between groups (*p* < 0.05).

EEG, electroencephalogram; T3.α1, T3 channel α1 wave; FP1.α2, FP1 channel α2 wave; T4. (θ+α2)/β1, T4 channel (θ+α2)/β1 value; T4.α1, T4 channel α1 wave; T4.α2, T4 channel α2 wave; T6.α2, T6 channel α2 wave; P4. (θ+α2)/β2, P4 channel (θ+α2)/β2 value; F4.α2, F4 channel α2 wave; P3. (θ+α1)/β2, P3 channel (θ+α1)/β2 value.

In order to compare the differences in physiological responses of university students to biological sounds at different stress levels (low, medium, and high), a detailed analysis of EEG was conducted. Under the stimulation of biological sounds, significant differences were observed in the α1 waves of P3 (*F*(2/349) = 4.39, *p* = 0.001, *η*^2^ = 0.03), P4 (*F*(2/349) = 3.88, *p* = 0.021, *η*^2^ = 0.02), and F3 (*F*(2/349) = 7.99, *p* = 0.001, *η*^2^ = 0.04), as well as the values of (θ+α2)/β2 of F7 (*F*(2/349) = 5.79, *p* = 0.001, *η*^2^ = 0.03). Post hoc testing (Tukey’s HSD) revealed that the α1 and α2 waves in each channel were the highest in the high-stress university, while the value of (θ+α2)/β2 was the lowest. This indicates that in the biological acoustic environment, university students under high stress have the highest sense of security and relaxation, and the alleviation of fatigue is also the most significant. Furthermore, for students with moderate and low stress levels, when they were exposed to biological sound stimuli, the α1 and α2 wave indices in the EEG of the moderate-stress-level students were higher than those of the low-stress-level students. Therefore, it can be concluded that in the biological sound environment, university students under greater stress feel safer and more relaxed. In terms of fatigue relief, the (θ+α)/β value of the EEG of students with lower stress is higher than that of students with moderate stress. For university students with moderate stress levels, the effect of biological sounds in relieving fatigue is better than that of students with low stress (Figures 1C and 1D).

### Effects of different biological sound sources

**Figure 2:**
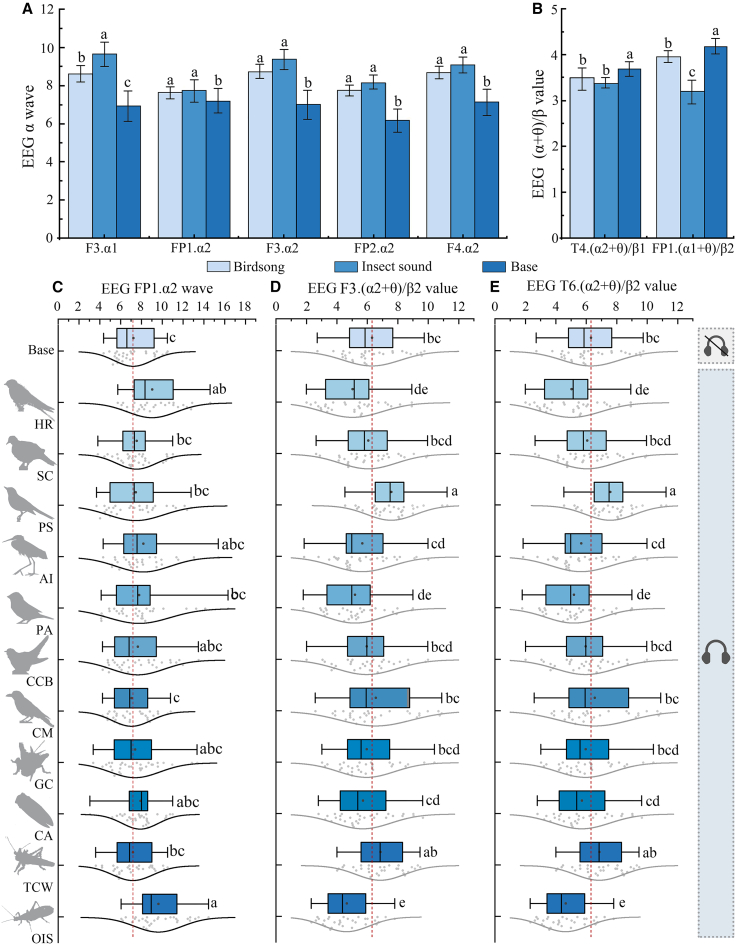
EEG responses of stressed university students to different types and kinds of biological sounds (A and B) Effects of listening to bird songs and insect sounds on EEG data. (C) Effects of 11 types of biological sounds on the EEG α2 wave. (D and E) Effects of 11 types of biological sounds on the EEG (α+θ)/β. (A and B) Bar graphs: mean ± Standard deviation. (C–E) Boxplots show mean (dot), median (line), 25th–75th percentiles (box), and mean ±1.5 Standard deviation (whiskers). Gray dots are individual data points. Lowercase letters (a, b) indicate significance, and different letters indicate significant differences between groups. F3. α2, F3 channel α2 wave; FP2. α2, FP2 channel α2 wave; FP1. (θ+α1)/β2, FP1 channel (θ+α1)/β2 value; HR, *Hirundo rustica*; SC, *Spilopelia chinensis*; PS, *Pica serica*; AI, *Ardea intermedia*; PA, *Passer ammodendri*; CCB, *Cuculus canorus bakeri*; CM, *Corvus macrorhynchos*; GC, *Gryllus chinensis*; CA, *Cryptotympana atrata*; TCW, *Tettigonia chinensis* Willemse; OIS, *Oecanthus indicus* Saussure*.*

When stimulated by the chirping of birds and the sounds of insects, the α1 waves of the F3 channel (*F* (2/381) = 3.98, *p* = 0.001, *η*^2^ = 0.02) and the α2 waves of the FP1 (*F*(2/381) = 3.99, *p* = 0.022, *η*^2^ = 0.02), F3 (*F* (2/381) = 3.81, *p* = 0.021, *η*^2^ = 0.02), FP2 (*F*(2/381) = 4.28, *p* = 0.001, *η*^2^ = 0.02), and F4 (*F*(2/381) = 2.96, *p* = 0.033, *η*^2^ = 0.02) channels in the brain were all higher than those at baseline rest, as shown in Figure 2A. The results show that both insects and birds can enhance the sense of security and relaxation of stressed university students (Table 3).

**Table 3:** Overview of biological sound stimulus samples

Biological sound types	Latin name	Abbreviation	Coordinate
Bird songs	*Hirundo rustica*	HR	113°42′46″E, 34°49′25″N
Bird songs	*Spilopelia chinensis*	SC	115°43′43″E, 34°35′17″N
Bird songs	*Pica serica*	PS	113°43′10″E, 34°49′14″N
Bird songs	*Ardea intermedia*	AI	115°42′42″E, 34°34′07″N
Bird songs	*Passer ammodendri*	PA	115°38′02″E, 34°33′38″N
Bird songs	*Cuculus canorus bakeri*	CCB	111°51′20″E, 34°20′20″N
Bird songs	*Corvus macrorhynchos*	CM	111°51′45″E, 34°19′01″N
Insect sounds	*Gryllus chinensis*	GC	113°42′40″E, 34°48′52″N
Insect sounds	*Cryptotympana atrata*	CA	111°51′05″E, 34°21′36″N
Insect sounds	*Tettigonia chinensis* Willemse	TCW	113°42′40″E, 34°49′17″N
Insect sounds	*Oecanthus indicus* Saussure	OIS	115°39′03″E, 34°34′05″N

The (α+θ)/β value is related to the fatigue that students experience under the stimulation of biological sounds. When students feel tired, this value increases significantly. As shown in Figure 2B, the baseline values of the T4 channel (α2+θ)/β1 (*F*(2/381) = 3.28, *p* = 0.032, *η*^2^ = 0.02) and the FP2 channel (α1+θ)/β2 (*F*(2/381) = 3.87, *p* = 0.021, *η*^2^ = 0.02) were significantly higher than those during the periods of bird chirping and insect sounds, indicating that when stimulated by biological sounds such as bird chirping and insect sounds, the fatigue of stressed university students was alleviated.

### Effects of different biological sound types

The α2 wave was related mainly to a relaxed mental state; the more relaxed the student was, the more active the α2 wave was. During the transition from the resting state to the auditory stimulation stage, stressed university students experienced varying degrees of changes in EEG waves, differences in the α2 wave in the frontal lobe FP1 channel (*F*(11/372) = 3.63, *p* = 0.001, *η*^2^ = 0.10) (Figure 2C). The frontal area is closely related to human memory, judgment, abstract thinking, and emotional behavior. The indices of the α2 wave at the FP1 channel decreased in the following order: *Oecanthus indicus* Saussure (OIS) > *Hirundo rustica* (HR) > *Ardea intermedia* (AI) > *Cryptotympana atrata* (CA) > *Passer ammodendri* (PA) > *Cuculus canorus bakeri* (CCB) > *Pica serica* (PS) > *Spilopelia chinensis* (SC) > *Gryllus chinensis* (GC) > Base > *Tettigonia chinensis* Willemse (TCW) > *Corvus macrorhynchos* (CM). The data showed that the stressed university students were most relaxed when listening to the sound of OIS, followed by HR and AI. However, the EEG indices for CM and TCW were lower than those for the base, indicating that these two types of biological sounds had a negative impact on the participants’ sense of relaxation during the process of analysis, memory perception, and mental thinking activities.

As shown in Figure 2, when transitioning from the resting state to the auditory stimulation stage, the university students’ F3. (α2+θ)/β2 (*F*(11/372) = 3.44, *p* = 0.001, *η*^2^ = 0.09) and T6. (α2+θ)/β2 (*F*(11/372) = 3.59, *p* = 0.001, *η*^2^ = 0.10) showed significant differences. The frontal lobe is mainly responsible for emotional processing; when students experience biological sound stimulation, there are eight kinds of biological sounds in the (α2+θ)/β2 value of EEG F3 that are lower than the baseline, and they are in turn (brain wave ratio from small to large) OIS < HR < PA < GC < CA < AI < CCB < SC. The EEG data showed that the stressed university students were affected to varying degrees in terms of fatigue when perceiving and processing these biological sounds; among them, OIS, HR, and PA have the most significant positive effects. The biological sounds with values lower than those in the base included PS, CM, and TCW, and these three types of biological sounds were more likely to cause fatigue in the students (Figure 2D).

The temporal lobe is related mainly to the understanding, memory, and mental activity of sounds and is also involved in the processing of emotions and social behavior. T6. (α2+θ)/β2 value data revealed that the stressed university students experienced an increased sense of auditory fatigue when stimulated by CM, PS, and TCW, and their values were higher than those in the base. However, auditory stimulation by HR, OIS, PA, CCB, AI, SC, and GC did not produce any sense of fatigue (Figure 2E).

### Psychological responses of biological sounds

In order to know the psychological reaction of participants after listening to biological sound, the same group of participants was selected, and after the EEG measurement experiment, they listened to biological sound again to complete semantic differential (SD) and Perceived Restorativeness Scale (PRS). The Cronbach’s α coefficients for the SD and PRS dimensions of the questionnaire were 0.87 and 0.90, respectively, both exceeding the threshold of 0.70. The overall Cronbach’s α coefficient for the questionnaire was 0.85, indicating good reliability and high internal consistency for both the subscales and the total scale.

The overall mean scores for bird songs were 3.00 ± 0.145 (SD) and 2.92 ± 0.13 (PRS), while for insect sounds they were 2.8 ± 0.58 (SD) and 2.71 ± 0.14 (PRS). A *t* test conducted on these comprehensive scores revealed significant differences in SD (*p* = 0.049, *t*(350) = 1.79, Cohen’s *d* = 0.19) and PRS (*p* = 0.050, *t*(350) = 1.93, Cohen’s *d* = 0.21) between exposure to bird songs and insect sounds, with both evaluations demonstrating higher scores for bird songs than for insect sounds. University students reported greater satisfaction with comfort (C), loudness (L), articulation (A), and preference (P) after listening to bird songs; simultaneously, bird songs exhibited superior performance in compatibility (CO), escape (ES), and extent (EX) compared to insect sounds.

A one-way ANOVA was subsequently performed on the 11 types of biological sounds, revealing significant differences in the composite scores of SD (*F*(10/341) = 17.24, *p* = 0.001, *η*^2^ = 0.35) and PRS (*F*(10/341) = 18.18, *p* = 0.001, *η*^2^ = 0.36) in the subjective questionnaires. Tukey’s HSD post hoc analysis demonstrated the following rankings for biological sound evaluations: HR > PA > OIS > AI > CA > SC > CCB > PS > GC > TCW > CM in terms of SD, and HR > OIS > PA > AI > PS > SC > CCB > CA > GC > TCW > CM for PRS. HR ranked highest in both dimensions, indicating its superior capacity to elicit positive psychological perceptions (Table 4).

**Table 4:** SD and PRS subscale scores

Biological sound	SD	PRS
HR	4.02 ± 0.16^a^	3.83 ± 0.15^a^
SC	2.73 ± 0.15^bcd^	2.63 ± 0.12^bcde^
PS	2.57 ± 0.14^cd^	2.72 ± 0.14^bcd^
AI	3.11 ± 0.17^bc^	3.04 ± 0.13^bc^
PA	3.71 ± 0.14^ab^	3.48 ± 0.17^ab^
CCB	2.66 ± 0.13^cd^	2.63 ± 0.12^bcde^
CM	2.2 ± 0.14^d^	2.08 ± 0.11^e^
OIS	3.69 ± 0.14^ab^	3.66 ± 0.12^ab^
GC	2.37 ± 0.09^d^	2.41 ± 0.12^cde^
CA	2.86 ± 0.17^cd^	2.62 ± 0.14^cde^
TCW	2.28 ± 0.18^d^	2.16 ± 0.18^de^

a-e Different lowercase letters (a-e) indicate significant differences between groups (*p* < 0.05).

SD, semantic differential; PRS, Perceived Restorativeness Scale.

### Exploratory analysis of psychological and physiological indices

**Figure 3:**
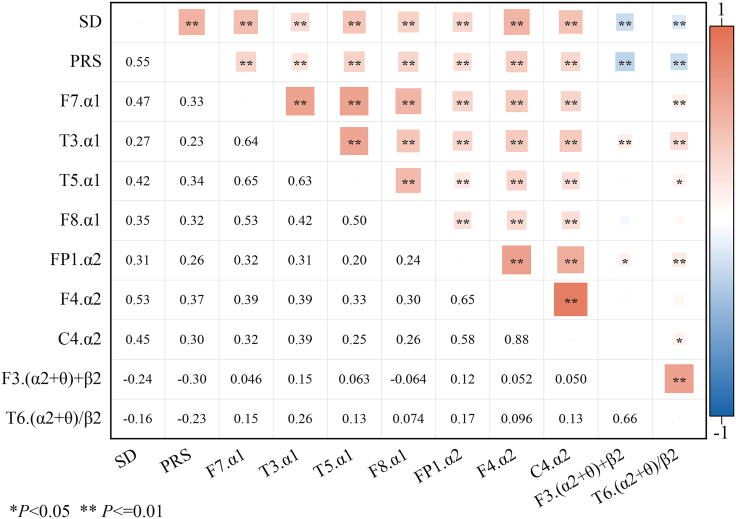
Correlation between psychological and physiological reactions F7.α1, F7 channel α1 wave; T5.α1, T5 channel α1 wave; F8.α1, F8 channel α1 wave; C4.α2, C4 channel α2 wave; F3.(θ+α2)/β2, F3 channel (θ+α3)/β2 value.

We explored the correlations among psychological and physiological responses, as illustrated in Figure 3. The questionnaire-derived psychological outcomes SD and PRS exhibited significantly positive correlations with the α1 waves in the EEG F7, T5, F8, and T3 channels (*p* < 0.001), as well as the α2 waves in the EEG FP1, F4, and C4 channels (*p* < 0.001). Conversely, negative correlations were observed between these psychological outcomes and the (α2+θ)/β2 values in the EEG F3 (*p* < 0.001) and T6 channels (*p* < 0.001). Notably, a strong positive correlation was identified between SD and the α2 wave in the F4 channel (*r* = 0.53, *p* < 0.001). Overall, the psychological and physiological responses of stressed university students under biological sound stimulation were interrelated. Higher amplitudes of EEG α1 and α2 waves corresponded to elevated scores in both SD and PRS, while increased (α+θ)/β values were associated with lower scores in these evaluations. These results clearly demonstrate an interdependent relationship between psychological and physiological responses.

By extracting the principal components from the predictor variable (X) and the response variable (Y), we obtained the principal components U and V, respectively, which acted as bridge factors in this study. The optimal quantity of principal components was determined through cross-validity analysis and variance inflation factor (VIF) measurement. The results show that the nine principal components of U and V effectively capture the relationship between the predictor variable and the response variable. A quantitative relationship model between EEG features and SD was established through partial least-squares regression (PLSR), and the regression equation shows SD = −0.188F3.(α2+θ)/β2 −0.108T6.(α2+θ)/β2 −0.036FP1.α2 + 0.416F4.α2 + 0.036C4.α2 + 0.277F7.α1 + 0.208T5.α1 + 0.036F8.α1 −0.157T3.α1. Standardized regression coefficients demonstrated that F4.α2 exerted the strongest positive effect on SD, while F3.(α2+θ)/β2 and T6.(α2+θ)/β2 contributed notably negative effect.

**Figure 4:**
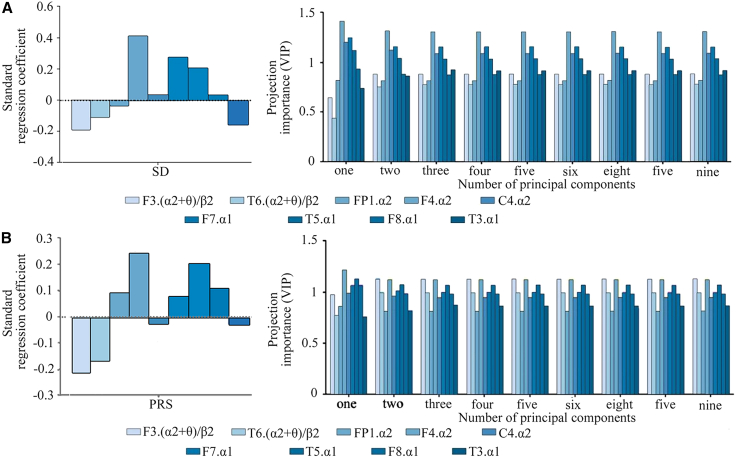
Standard regression coefficient and projection importance index (VIP) (A) SD and EEG. (B) PRS and EEG.

In the accuracy analysis, the interpretation rate of electroencephalogram characteristic variations was 11.1%. The explanation rate of SD variation was 59.3%. These findings underscore the importance of V in capturing the variability of the dependent variable. Furthermore, the variable importance in projection (VIP) analysis revealed an increase in the VIP values of F4.α2 (VIP = 1.420) and C4.α2 (VIP = 1.208), highlighting their substantial importance in predicting the dependent variable and explaining its characteristics (Figure 4A).

By extracting the principal components of the independent variable X and the dependent variable Y, the principal components U and V were obtained, serving as the bridge of this study. During the extraction process, cross-validity analysis and VIF indicators were used to determine the optimal quantity of principal components. The results show that both U and V contain nine principal components, effectively capturing the relationship between the independent variable and the dependent variable. A quantitative relationship model between EEG features and PRS was established through PLSR. The regression equation shows PRS = −0.214F3.(α2+θ)/β2 −0.166T6.(α2+θ)/β2 + 0.090FP1.α2 + 0.242F4.α2 −0.025C4.α2 + 0.077F7.α1 + 0.203T5.α1 + 0.106F8.α1 −0.029T3.α1. The standardized regression coefficients demonstrated that F4.α2 exerted the strongest positive effect on PRS, while F3.(α2+θ)/β2 and T6.(α2+θ)/β2 exhibited notable negative influences.

In the accuracy analysis, the interpretation rate of electroencephalogram characteristic variations was 11.1%, and the interpretation rate of SD variation was 71.3%. This indicates that V is significantly more effective in explaining the variance of Y. Furthermore, the VIP analysis revealed that the VIP values of F4.α2 (VIP = 1.220) and T5.α1 (VIP = 1.141) increased, highlighting their substantial importance in explaining the dependent variable Y (Figure 4B).

**Figure 5:**
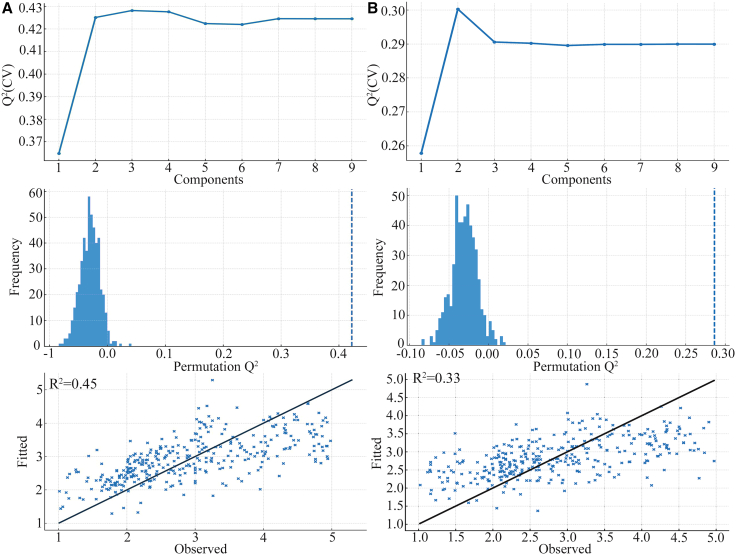
Cross-validation and permutation tests for PLSR models (A) SD model: (upper) cross-validated Q^2^ versus the number of latent components; (middle) histogram of Q^2^ values from 500 permutation tests; the red vertical line indicates the Q^2^ of the true model; (below) correlation between observed and model-fitted values. (B) PRS model: the same set of validation plots as in (A).

To assess the robustness and statistical significance of the established PLSR model, we conducted 10-fold cross-validation and a Y permutation test (B = 500). Based on the RMSECV + one-SE rule, the optimal latent score for both models was 2. The SD model demonstrated excellent predictive power (Q^2^ = 0.43), while the PRS model had an acceptable predictive ability (Q^2^ = 0.31) (Figures 5A and 5B). The permutation results showed that the observed Q^2^ values of both models were significantly higher than the permutation distribution (*p* < 1/(B + 1) = 0.002), suggesting that the association between EEG features and psychological responses was not accidental.

### Demographic characteristics

**Figure 6:**
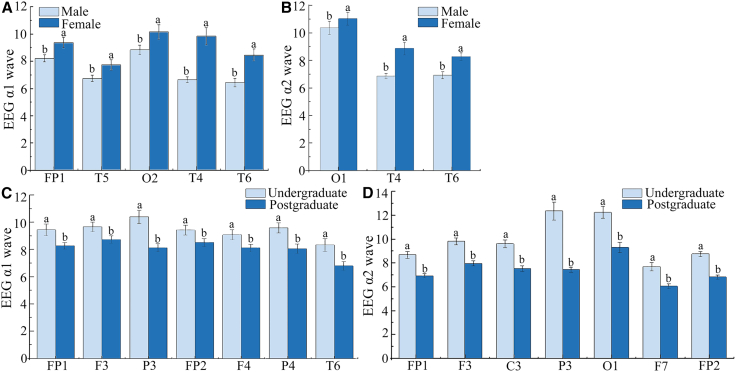
EEG responses of different genders and education levels of stressed university students to biological sounds (A) Different genders EEG α1 wave. (B) Different genders EEG α2 wave. (C) Different educational background EEG α1 wave. (D) Different educational background EEG α2 wave. Data are presented as mean ± Standard deviation. Lowercase letters (a, b) indicate significance, and different letters indicate significant differences between groups.

Analysis of the α1 wave comparison between male and female students showed significant differences at FP1 (*p* = 0.001, *t*(307) = −3.15, Cohen’s *d* = 0.34), T5 (*p* = 0.030, *t*(350) = −2.20, Cohen’s *d* = 0.2), T6 (*p* = 0.001, *t*(346) = −3.17, Cohen’s *d* = 0.2), T4 (*p* = 0.001, *t*(220) = −5.52, Cohen’s *d* = 0.74), and O2 (*p* = 0.001; *t*(321) = −3.53; Cohen’s *d* = 0.39). The α1 wave under biological sound stimulation was higher in females than in males, indicating that males respond less to biological sounds than females do and that females are more likely to relax or feel secure in environments with biological sound. The α2 wave showed significant differences at O1 (*p* = 0.026, *t*(349) = −2.24, Cohen’s *d* = 0.24), T4 (*p* = 0.001, *t*(229) = −5.18, Cohen’s *d* = 0.69), and T6 (*p* = 0.001, *t*(346) = −4.67, Cohen’s *d* = 0.34), which was mainly related to the relaxed mental state; the happier the mood was, the more active the α2 wave was. In the α2 wave, the EEG index of females was higher than that of males, indicating that females experience greater relaxation in response to biological sounds than males do (Figures 6A and 6B).

By comparing the EEG wave data of participants with different educational backgrounds, we observed significant differences in the α1 wave at the FP1 (*p* = 0.001, *t*(284) = 4.34, Cohen’s *d* = 0.52), F3 (*p* = 0.001, *t*(256) = 4.23, Cohen’s *d* = 0.53), FP2 (*p* = 0.001, *t*(286) = 4.74, Cohen’s *d* = 0.56), F4 (*p* = 0.001, *t*(333) = 4.10, Cohen’s *d* = 0.45), P3 (*p* = 0.001, *t*(297) = 5.47, Cohen’s *d* = 0.63), P4 (*p* = 0.001, *t*(346) = 4.81, Cohen’s *d* = 0.52), and T6 (*p* = 0.001, *t*(327) = 4.42, Cohen’s *d* = 0.49) channels. The α1 wave associated with a sense of security and relaxation was higher than that of graduate students in all channels, indicating that undergraduate students responded more to biological sounds than graduate students. Undergraduate students were more likely to relax or feel safe in an environment with biological sound. The α2 wave showed significant differences in the FP1 (*p* = 0.001, *t*(314) = 10.78, Cohen’s *d* = 1.22), F3 (*p* = 0.001, *t*(295) = 9.22, Cohen’s *d* = 1.07), P3 (*p* = 0.001, *t*(207) = 7.98, Cohen’s *d* = 1.11), C3 (*p* = 0.001, *t*(294) = 7.88, Cohen’s *d* = 0.92), O1 (*p* = 0.001, *t*(349) = 6.11, Cohen’s *d* = 0.65), F7 (*p* = 0.001, *t*(280) = 6.77, Cohen’s *d* = 0.81), and FP2 (*p* = 0.001, *t*(281) = 10.30, Cohen’s *d* = 1.23) channels, and the differences were higher in undergraduate students than in graduate students. This indicates that undergraduate students feel more relaxed in response to biological sounds than graduate students (Figures 6C and 6D).

## Discussion

### Stressed university students can obtain positive effects in environments with biological sounds

This study, through its specific design, has achieved the deepening and supplementation of existing research. In terms of participant selection, this study focused specifically on the group of stressed-out university students, making up for the limitations of existing research in its attention to the general population. By combining “EEG measurement + questionnaire psychological perception” as a research method, it was confirmed that exposure to biological sounds can significantly enhance their sense of security and relaxation and reduce fatigue. It also broke through the traditional approach of treating biological sounds as a homogeneous whole and deeply analyzed the specific effects of different sound sources and biological species. Among them, the influences of OI, HR, CA, AI, PA, CCB, and SC were the most significant. This reinforced and expanded the existing research on the positive effects of natural soundscapes. This indicates that bird songs and other biological sounds are suitable types of natural sounds for releasing stress and restoring attention and have significant potential for stress recovery.^55^ This result is consistent with the theoretical frameworks of SRT^17^ and ART.^15^ These theories suggest that natural stimuli with positive attributes can induce physiological relaxation, alleviate stress, and restore depleted cognitive resources. This is because biological sounds can trigger unconscious positive emotions such as a sense of security and natural connection, interrupting stress-related cognitive rumination^35^^,^^56^^,^^57^ and significantly reducing fatigue and anxiety. This is precisely the core mechanism emphasized by SRT.^32^ ART further clarifies the unique effectiveness of biological sounds—their “soft allure” (such as rhythmic bird songs and gentle insect chirps) can activate the unconscious attention, allowing the focused attention system depleted by mundane demands to rest,^58^ which is consistent with the conclusion proposed by Wang et al. that “natural sound stimulation promotes perceptual harmony and reduces stress through sensory and cognitive pathways.”^37^ Furthermore, Liu’s research also indicates from a physiological perspective that bird songs and other biological sounds can inhibit the excessive activation of the sympathetic nervous system, manifested as an increase in alpha wave power (related to relaxation).^59^ However, Wang et al. used changes in breathing frequency to find that the influence of insect chirping, bird songs, and other biological sounds on breathing frequency is overall very small, far less than that of earth-based sounds such as water sounds.^37^ Based on the research conclusions of Bradley, Gomez, and Danus-er, this might be because the influence of the sound environment on breathing frequency could be related to the volume of the sound and its emotional elements.^60^ This requires further verification through expanding the research subjects in future studies.

Furthermore, through quantitative analysis of EEG, it was found that under the stimulation of biological sounds, university students at different stress levels exhibited different physiological responses on the EEG. The higher the score on the stress perception scale, the greater the stress the students were experiencing, and the more significant the positive impact of the biological sounds was. This is an important finding of this study. This might be because the physiological systems of individuals under high stress are often in a baseline state of heightened sympathetic nerve arousal,^61^ and their physiological conditions deviated from equilibrium provide greater regulatory space for biological sounds to trigger a parasympathetic nerve-dominated relaxation response,^35^ thus resulting in more significant changes. Furthermore, students under high pressure often suffer from more severe attention fatigue and depletion of cognitive resources,^62^ and the “soft charm” of biological sounds can effectively attract their unconscious attention,^58^ thereby freeing them from repetitive thoughts related to stress and demonstrating a more positive impact.

In conclusion, biological sounds, as an effective stress intervention method, have significant application value for individuals with high stress levels, further demonstrating the importance of integrating biological sounds into environments that need to reduce stress (such as university campuses). When designing urban green spaces or campus landscapes, merely focusing on vegetation and water bodies to support diverse wildlife (thus generating various biological sounds) is not sufficient; purposefully and meticulously planning the acoustic characteristics may maximize the restorative effect.

### Comparison of physiological reaction and psychological feeling of stressed university students

**Figure 7:**
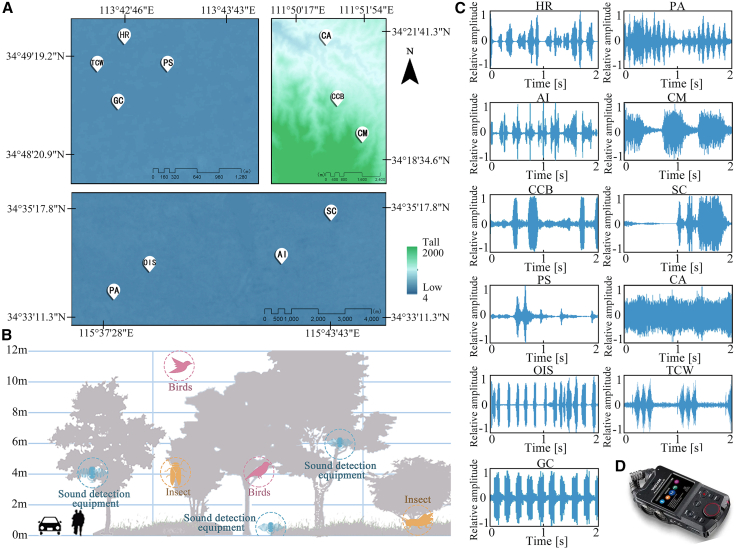
Biological sound acquisition equipment, location, and amplitude (A) Locations of the 11 types of biological sounds. (B) Height of sound monitoring equipment placement and distribution of biological sounds. (C) Biological sound waveforms. (D) Portable audio field recorder (TASCAM Portacapture X8).

The internal linkage between psychological responses and brain physiological indicators of stressed university students under biological sound stimulation is established through correlation analysis, and an overall correlation framework is constructed. Then, the PLSR is used to quantify the key influencing factors, providing empirical support for the physiological-psychological linkage mechanism and deepening the systematic understanding of the biological sound regulation mechanism of stress. Overall, the EEG results of a single source of biological sound have a high degree of consistency with psychological perception: HR, PA, OIS, and CA are the stimulus types with the most significant positive recovery effects. CM and TCW, however, consistently show negative impacts in both evaluations. This is highly consistent with previous research results, in which “electrophysiological measurement of brain activity + psychological perception questionnaire” has a high degree of correlation,^54^ and also proves the reliability of physiological data. However, there are also clear discrepancies between the EEG results and the psychological questionnaire results. The EEG findings indicate that insect chirping is more effective than bird chirping in enhancing feelings of security, pleasure, and reducing fatigue. The psychological questionnaire, on the other hand, leans more toward bird chirping. The questionnaire results are consistent with the conclusion of Liang et al. that “the subjective positive effect of insect chirping is weaker than that of bird chirping.”^63^ This result may be the result of multiple factors working together. Insect chirping is mostly high-frequency rhythmic sounds ranging from 2 to 20 kHz, which may evoke physiological arousal in humans.^37^ In contrast, the chirping of birds is produced by the vibration of air flow in the larynx and trachea, which falls within a lower frequency range and has a higher degree of variability and melodiousness, making it more likely to capture the interest of the subjects.^64^ Additionally, the advantage of insect calls is largely driven by OIS. Without OIS, the vocalizations of birds would have a greater advantage in the local entertainment culture of Henan (Figure 7).

Furthermore, the impact of the experimental design cannot be ruled out. In this research, to ensure the reliability of the data on physiological responses and psychological feelings, three safeguards were implemented: first, there was an informed consent process and an unconditional withdrawal mechanism before the experiment, which reduced the response bias caused by participants’ “passive participation.” Second, a 1-h time interval enables a transition to a more stable “real state.”^65^^,^^66^ At this point, the psychological assessment can more accurately correspond to the core neural activities reflected by the EEG recordings. The third is the mature paradigm within the domain of psychological measurement of acoustic scenes,^67^^,^^68^ which satisfies the internal consistency reliability criteria. We acknowledge that this cannot entirely rule out the potential influence of confounding factors in the experimental design. However, the presence of these potential confounders does not invalidate the current findings; rather, they hold significant value by proposing a testable hypothesis for future research—specifically, the “optimal temporal matching threshold for physiological and psychological data under bioacoustic stimulation conditions.”

### Physiological response of stressed university students to biological sounds is affected by demographic characteristics

The results show that sex significantly affects people’s perception of biological sounds. There are significant differences in the feelings of pleasure and security between males and females when exposed to biological sounds. This is consistent with previous research results. Sex influences the evaluation of biological sounds, especially in terms of feelings of pleasure and so on,^69^ but this study verified Fang’s conclusion through physiological responses. The EEG results show that females have a more positive response to biological sounds, with greater feelings of security and relaxation. This is consistent with the viewpoints stated in the literature, which suggest that females may be more likely to express positive emotions in a restorative environment,^42^ and soothing sounds are particularly effective in relieving their stress.^40^ These differences also reflect the different cognitive processing strategies of male and females. Females tend to adopt a “top-bottom” approach, linking personal meaning and emotional resonance with the environment, while males exhibit a “bottom-top” style, relying more on direct sensory input.^70^

Educational level significantly moderates the physiological responses of individuals when exposed to biological sounds. The EEG results of undergraduate students showed a stronger sense of security and relaxation, which can be confirmed from the higher α1 and α2 waves in all channels they exhibited. This discovery is in line with the view that “educated individuals tend to have more critical thinking skills in environmental assessments,”^71^ which might make undergraduate students feel more content and relaxed in such a quiet environment.^72^ Therefore, although the physiological responses of graduate students were more intense, the subjective recovery benefits obtained by undergraduate students were more significant.

### Limitations of the study

The positive effects of biological sounds are not constant but are the complex result of the dynamic characteristics of the sound environment itself and the individual characteristics of the participants. At the environmental level, forest soundscapes show significant seasonal variations, and the same biological sounds may elicit different auditory experiences and reactions in different seasons.^73^ At the individual level, factors such as age, life experiences,^59^ and familiarity with sounds^69^ all influence how people perceive biological sounds. Therefore, in the future, based on these findings, a perspective spanning different age groups will be incorporated, and longitudinal tracking across seasons will be carried out. It is crucial to include an assessment of the familiarity of the participants’ voices and personal connections, so as to more accurately reveal the psychological and physiological mechanisms by which biological sounds affect the human body and mind.

**Figure 8:**
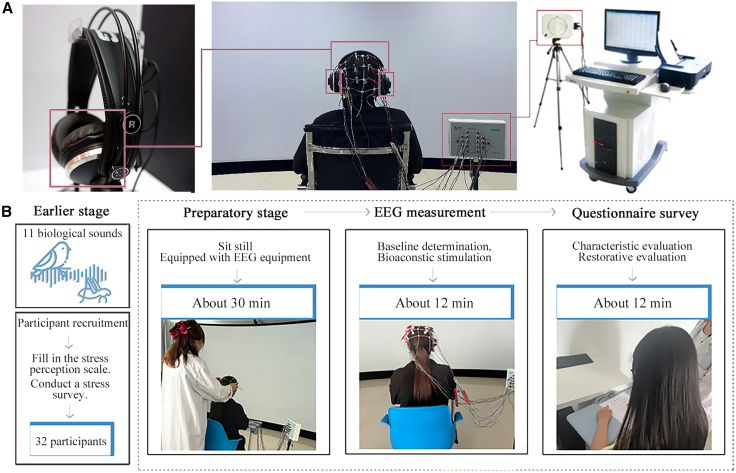
Experimental equipment and process (A) Sound playing equipment (AKG K271MKII) and EEG equipment (Novin ND-97). (B) Experimental process.

Furthermore, the issue of the “1-h interval” between physiological and psychological measurements in the current experimental design warrants further investigation (Figure 8). Our experiment was based on Hwang’s research,^65^ which indicated that “EEG readings can reflect optimal emotional states after 1 h of rest.” However, upon further analysis, we cannot help but consider the following questions: might participants potentially recover within a shorter time frame than 1 h? or would longer time intervals produce positive or negative effects? It is hoped that future research will examine the correlation between fatigue and cumulative effects of physiological and psychological measurements under different durations (such as 10 min, 30 min, and 1 h).

## Resource availability

### Lead contact

Further information and requests for resources should be directed to and will be fulfilled by the lead contact, Jie Zhang (zhangjie0421@beihua.edu.cn).

### Materials availability

This study did not generate new unique reagents.

### Data and code availability

• Data reported in this paper have been deposited at Zenodo (https://doi.org/10.5281/zenodo.17535641) and will also be shared by the lead contact upon request.
• Original code has been deposited at Zenodo (https://doi.org/10.5281/zenodo.17535641) and is publicly available.
• Any additional information required to reanalyze the data reported in this paper is available from the lead contact upon request.

## Acknowledgments

We sincerely thank the staff of the Administrative Office of Zhengzhou National Forest Park, Shangqiu Yellow River Old Course National Forest Park, and Luoyang Huaguoshan National Forest Park for their support and assistance during the process of biological sound collection.

This research was funded by the National Natural Science Foundation of China Project (grant no. 32572152), the Natural Science Fund of Jilin Province Science and Technology Department (grant no. YDZJ202501ZYTS534), the Natural Science Research Project of Jilin Provincial Department of Education (grant no. JJKH20250815KJ), the Private Fund Project of Beihua University (grant no. 320121060), and the National Landscape Architecture Professional Degree Postgraduate Education Steering Committee (grant no. LAJGXM2025059).

## Author contributions

Conceptualization, T.H. and J.Z.; methodology, T.H., J.Z., and Y.Z.; investigation, T.H., X.P., T.S., and W.G.; writing – original draft, T.H. and J.Z.; writing – review and editing, A.Z. and C.Z.; funding acquisition, J.Z.; resources, J.Z. and T.H.; supervision, J.Z. and S.S.

## Declaration of interests

The authors declare no competing interests.

## STAR★Methods

### Key resources table

**Table:** 

REAGENT or RESOURCE	SOURCE	IDENTIFIER
**Deposited data**		

Original data	This paper	https://doi.org/10.5281/zenodo.17535641

**Software and algorithms**		

SPSS Statistics ver. 27	IBM	https://www.ibm.com/products/spss-statistics
eegUtils package	CRAN	https://craddm.github.io/eegUtils/index.html
Python (version 3.9.5)	Python Software Foundation	https://www.python.org/
R version 4.4.4	R Core Team	https://www.r-project.org/
G∗Power 3.1.9.7	Heinrich-Heine-Universität Düsseldorf	https://www.psychologie.hhu.de/arbeitsgruppen/allgemeine-psychologie-und-arbeitspsychologie/gpower
Adobe Audition 2021	Adobe Inc.	https://www.adobe.com

**Other**		

Biological sound	Forest park collection	https://doi.org/10.5281/zenodo.17535037

### Experimental model and study participant details

As research has shown that university students as experimental participants have broad knowledge and a scientific nature,^56^ participants aged 23±1.8 years were recruited from university campuses. Due to the particularity of EEG experiment^54^ and the requirements of the experiment, participants were required to be right-handed, in good physical and mental health, with normal hearing (0-24 dB HL) and without a history of mental illness. Moreover, they were not allowed to consume alcohol or drugs three days before the experiment. The author states that all experiments comply with the ethical standards of the Institutional Review Board for Human Experiments, particularly the Helsinki Declaration (revised in 2008) (1975). This experiment has been approved by the Medical Ethics Committee of Beihua University Hospital (ERC-2022-047), and all participants voluntarily signed the informed consent form. Staff members explicitly informed participants of key information such as the core nature of the experiment, its general procedure, and the total time required. They also explicitly stated that: if participants felt any physical discomfort or emotional distress at any stage of the experiment, they could unconditionally choose to withdraw from the experiment. To minimize interference from visual stimuli on the results, all participants were required to wear eye masks during the experiment.

To assess the students’ stress levels before the experiment, the participants completed the stress perception scale before the biological sound stimulation experiment. The stress perception scale was developed by Cohen et al.^53^ to detect overall and general stress in an individual's life one month before the use of biological sound stimulation, which represents a degree of self-awareness. The stress perception scale consists of 14 items, including the two dimensions of tension (e.g., “I often feel overwhelmed by the accumulation of difficulties that I cannot overcome”) and a sense of being out of control (e.g., “I often get angry because many things happen beyond my control”). The dimension of loss of control consists of 8 items, with a Cronbach’s α coefficient of 0.86; the dimension of tension consists of 6 items, with a Cronbach’s α coefficient of 0.90. The α coefficients for both dimensions of the PSS scale exceeded the critical threshold of 0.70, indicating good internal consistency reliability for each dimension. The overall Cronbach’s α coefficient for the scale was 0.93, demonstrating a high level of overall reliability. We selected university students who experienced perceived stress as the research subjects. Participants voluntarily participated in the experiment and met the stress criterion of PSS score ≥ 18 as mentioned in the relevant literature.^74^ To further explore the regulatory effect of stress intensity on the biological sounds response, and to facilitate the inter-group statistical comparison in the subsequent EEG experiment, the grouping was conducted using an operational definition based on the distribution within the sample^75^: All the participants were sorted by their PSS scores from the lowest to the highest, and then divided into three groups: the low-stress group (18 ≤ score ≤ 30), the moderate-stress group (31 ≤ score ≤ 38), and the high-stress group (score ≥ 39). This grouping method is widely used in stress assessment studies of non-clinical university students, which can ensure the balanced sample size distribution among the three groups and effectively control the interference of extreme values on the statistical test power.

Participant recruitment information was released via the social media platforms of Beihua University. Using G∗Power 3.1.9.7 software to calculate the minimum sample size, the parameters were set as follows: effect size f = 0.25 (medium), α = 0.05, statistical power = 0.80, number of measurements = 11, correlation among repeated measures = 0.5, and correction for nonsphericity ε = 1. Finally, a total of 32 participants have been recruited in combination with relevant studies.^54^^,^^76^ Post-event statistical analysis (chi-square test) revealed that there were no significant differences in sex (*χ*^2^ = 4.58, *P* = 0.11), educational background (*χ*^2^ = 1.31, *P* = 0.52), and background educational attainment (*χ*^2^ = 3.04, *P* = 0.22) among the different stress groups. This indicates that these variables were well balanced across the groups (Table 1). The influence of sex on participant responses was systematically examined in the Demographic characteristics section of the Results.

### Method details

#### EEG parameters

EEG have good temporal resolution and can monitor real-time emotional processes caused by exposure to external environmental stimuli.^77^ Their application in current landscape research is mainly in health landscape and restorative research, landscape attention and preference research, and landscape psychological fatigue and audiovisual fatigue research. EEG have also become an important method for landscape evaluation research, building a technical bridge for “environment–psychology” research in landscapes.^59^ The frontal lobe is located in front of the brain, including the forehead (left: FP1, right: FP2) and front pole (left: F3, right: F4); the parietal lobe is located on the top of the brain, including the central area (left: C3, right: C4) and the parietal area (left: P3, right: P4); the occipital area (left: O1, right: O2) is located at the back of the brain; and the temporal lobe is located on both sides of the brain, including the front temporal (left: F7, right: F8), middle temporal (left: T3, right: T4), and rear temporal (left: T5, right: T6).^72^

At present, EEG often use multiple electrodes connected to the scalp surface for synchronous recording, including α, β, δ, and θ (4-8 Hz) wave frequency components. Among them, α and β waves are more suitable indicators for exploring differences in sound environments.^49^ The α and β waves are subdivided into α1 waves (8-10 Hz), α2 waves (10-13 Hz), β1 waves (13-20 Hz), and β2 waves (20-30 Hz) as more refined evaluation indicators to determine the brain's response to biological sounds under different conditions. The corresponding brain states are α1 waves, which signify a sense of security, relaxation, and unguardedness in the surrounding environment; α2 waves, which indicate a lack of pressure and anxiety, relaxation, pleasure, and alertness^49^^,^^72^; α waves, which are of low frequency and are produced at a low level of physiological arousal; β waves, which are of higher frequency and are produced when a person is alert, focused, and efficient; and θ waves, which indicate a state of deep sleep and unconsciousness. When a person is in a state of fatigue, the brain's thinking activity decreases, thus reducing β waves and high-frequency EEG, whereas α waves increase; when a person transitions from fatigue to drowsiness or sleep, the dominant EEG frequency gradually decreases to slow θ waves. Therefore, (α+θ)/β value can be used to describe the EEG characteristics of subjects' fatigue, and it can be used as an indicator of the influence of sound sources on fatigue.^50^

#### Soundscape psychological questionnaire

In order to understand the psychological participants after listening to biological sounds, the same group of Stressed University Students were selected. After the EEG measurement experiment, they listened to biological sounds again to complete two evaluations: (1) SD and (2) PRS. To ensure validity, the original English scales underwent a standard translation and back-translation process to produce semantically equivalent Chinese versions. These multidimensional metrics, thus adapted from analogous study designs,^78^ enabled the integrative analysis of biological sound properties, restorative effects, and neural correlates.

The SD method was implemented to assess participants' perceptual evaluations of the biological sounds, following established practices in soundscape research.^79^^,^^80^ This technique allows for the systematic identification of salient acoustic features. The SD comprised four indicator: Comfort level (C), Articulation (A), Loudness (L), and Satisfaction (S).^67^

PRS quantified restorative environmental properties that replenish depleted cognitive and physiological resources,^81^ structured across three indicator, Compatibility (CO)Auditory non-interference with intended site-specific activities , Escape (ES) Perceived liberation from occupational/responsibility stressors, Extend (EX)Attention diversification and extended activity engagement through sound-mediated cognitive associations.^68^^,^^82^ All items were scored via a 5-point Likert scale (1: Almost none; 2: Minimal; 3: Moderate; 4: Substantial; 5: Extensive).

(Equation 1) SD=(C+A+L+S)/4

Among them, C represents comfort level score, A signifies escape score, L denotes loudness score, P indicates preference score, and SD corresponds to total characteristic evaluation score.

(Equation 2) PRS=(CO+ES+EX)/3

Among them, CO represents compatibility score, ES signifies Escape score, EX indicates Extend score and PRS denotes total perceived restorativeness score.

#### Bioacoustic collection and selection

Henan Province is located in the central and eastern parts of China, in the middle and lower reaches of the Yellow River. Its terrain as a whole presents a pattern of “high in the west and low in the east, surrounded by mountains on three sides, and vast plains in the middle and east”. Based on the geographical and spatial characteristics of Henan Province, the typical sampling method was adopted to screen out three representative national forest parks: Zhengzhou National Forest Park, National Forest Park of the Old Yellow River and Huanguo Mountain National Forest Park (Figure 7A).

Forest creatures are most active from June to September. Clear weather with light wind and little fog was chosen for the passive monitoring and collection of biological sounds. Before collection, research was conducted to collect, count, and verify the main birds and insects in Henan Province, with the aim of selecting biological sounds familiar to local tourists for acquisition. During collection, two people worked in parallel, carrying a sound level metre (BSW A801) and a high-fidelity recorder (TASCAM Portacapture X8) (Figure 7D). The biological sounds were recorded in three parks in an alternating manner. The recording equipment was set to continuously record for 30 minutes, followed by a 30-minute pause. Each sampling point was monitored continuously for 2 days, and then the collection was repeated once after an interval of one month.

Background noise levels were verified with the BSW A801 sound level meter to remain below 45 dB (LAeq), thus ensuring minimal persistent anthropogenic noise and high recording integrity. The recorders were positioned at a standardized distance of 0.5 m from primary biological activity sites to reduce ambient acoustic interference. Audio was acquired at a sampling rate of 44.1 kHz, with 16-bit depth and stereo channel configuration, and saved in uncompressed WAV format to preserve original acoustic characteristics (Figure 7B). The entire field recording methodology, including equipment selection, parameter setting, and environmental control, adhered to the guidelines outlined in ISO/TS 12913-2:2018 for soundscape data collection.

After the completion of bioacoustic audio collection, the data were cut every 15 minutes to extract a 45s biological sound sample, filtered out clips containing highly repetitive time-frequency patterns or significant background noise interference to ensure high-fidelity, target-specific sounds. The sounds of 11 common creatures in the forest park were ultimately selected as research samples, including 7 types of birds and 4 types of insects (Table 3). To study the impact of a single source of biological sounds on humans, Adobe Audition 2021 sound processing software was used to extract and denoise the intercepted biological sounds to ensure that the participants in the experiment received only one type of biological sounds stimulus (Figure 7C). Finally, for the biological sounds playback, all stimuli were standardized to a duration of 40 seconds.^83^ Their sound pressure level (SPL) was maintained at a stable and consistent level (50 dB).^59^ At the same time, their original frequency characteristics were retained.

#### Experimental procedure and stimuli

The experiment was conducted in the Virtual Key Laboratory at Beihua University (Figure 8A). Throughout the experimental sessions, temperature, relative humidity, and illuminance were maintained at 22°C, 50%, and 500 lux, respectively. EEG signals were acquired using a 16-channel NOVA KJ-2000 EEG topographic mapping system. The data were sampled at 100 Hz. During the online recording, the bilateral earlobes (A1 and A2) served as the reference. Auditory sounds were delivered via AKG K271MKII headphones with a frequency response range of 16–28,000 Hz and sensitivity of 91 dB/m.

The experiments were conducted between 10:00 AM to 12:00 PM and 2:00 PM to 6:00 PM, comprising four sequential phases: Preparation, Baseline, EEG Experimental Measurement, and Questionnaire Survey, as illustrated in Figure 8B. (1) Preparation Phase: Participants sat quietly for 30 minutes to eliminate environmental carryover effects.^59^ During this period, they were briefed on the general procedure and fitted with physiological monitoring equipment. (2) EEG Experimental Measurement Phase: To evaluate the impact of biological sounds on participants, a 40-second baseline interval (devoid of auditory, visual, or other sounds) was first established. Participants then wore headphones to receive distinct biological sounds stimuli. Each biological sounds lasted 40 seconds, with 20-second intervals between sounds, while EEG signals were continuously monitored. The duration of the biological sounds stimulation and the intervals were selected based on the related research of soundscapes EEG.^84^ At the same time, considering the nature of biological sounds, a longer time was adopted to ensure sufficient time for immersion and neural recovery. (3) After the EEG measurement, the participants were arranged to rest in a quiet laboratory for one hour to minimize any potential residual effects during the physiological measurement stage.^65^^,^^66^ Following this break, the biological sounds were replayed. Immediately thereafter, the participants filled out a psychological questionnaire to evaluate their subjective experiences. During the administration of the questionnaire, the sound played for each participant was randomly selected and did not align with the sequence of the EEG measurements.

The sequence of biological sounds was randomized, and a Latin square designwas employed to control order effects. Based on this matrix, the 32 participants were allocated to balance exposure to distinct biological sounds and mitigate sequence effects.^54^ Post-event statistical analysis confirmed that there was no significant difference in the distribution of the stress group across the Latin square sequence (*χ*^2^ = 21.92, *P* = 0.35). Prior studies on physiological and emotional responses to auditory stimuli indicate that instantaneous auditory stimuli exceeding 2 seconds can evoke measurable emotional changes, detectable via physiological indices.^85^ To prevent participant fatigue caused by prolonged EEG acquisition, total measurement durations must be limited to 15 minutes. Accordingly, each biological sounds was set to 40 seconds to balance detectability and protocol feasibility.^84^

### Quantification and statistical analysis

The electroencephalogram (EEG) comprises mixed waveforms of amplitude and frequency, categorized into six distinct waves based on spectral characteristics. Relative power of preprocessed EEG data was calculated using the wavelet transform method in the MATLAB EEG toolbox, segmented by characteristic waveforms across frequency waves. It is worth noting that although the EEG signals were recorded from all 16 channels that provide complete scalp coverage, the channels listed in the paper are those brain electrical channels that showed significant results after analyzing all the channels. Each topographic map represents the averaged EEG power across all 32 participants, with red regions indicating positive amplitude and blue regions denoting negative amplitude. We use the function topoplot in the eegUtils package (https://craddm.github.io/eegUtils/index.html) of software R 4.4.4 for visualization.These maps visually elucidated the neurophysiological effects of biological sounds on stressed university students.^86^

To systematically compare the effects of diverse biological sounds on psychological and physiological responses, the no-stimulation phase was defined as the baseline condition in this study. All electroencephalogram (EEG) and questionnaire data were processed and analyzed with SPSS 27 software. The Shapiro-Wilk test was first employed to evaluate the normality of data distributions. Subsequently, one-way analysis of variance (ANOVA), independent samples t-tests, and least significant difference (Tukey HSD) post hoc analyses were conducted to identify intergroup variations. Spearman’s rank correlation test was applied to assess relationships between measured parameters, while partial least squares regression (PLSR) was utilized to interpret the variance between maximal psychological and physiological responses. Statistical significance was determined at (*P* < 0.05 ). In boxplots, the interquartile range (central 50% of data) is depicted by box boundaries, with whiskers extending to extreme values within the normal range. The median is represented by a horizontal line within the box, and the mean is indicated by a dot. Scatter points adjacent to boxes display individual data from 32 participants, and overlaid curves illustrate the normative distribution of the dataset.

Baseline measures differ significantly among populations with divergent stress levels and demographic attributes. Therefore, quantifying relative changes is essential to analyze the differential impacts of biological sounds across these groups (3):

(Equation 3) RCV(%)=(ABAS−Base)/Base

(RCV: Relative change value; ABAS: After biological acoustic stimulation).

## Supplemental information

Data S1. Ethics approval document from the Medical Ethics Committee of Beihua University Hospital (ERC-2022-047)

## References

[1] Hsieh C.H., Yang J.Y., Huang C.W., Chin W.C.B. (2023). The effect of water sound level in virtual reality: A study of restorative benefits in young adults through immersive natural environments. J. Environ. Psychol..

[2] Dinger M.K., Vesely S.K. (2001). Relationships between physical activity and other health-related behaviors in a representative sample of US college students. Am. J. Health Educ..

[3] He M., Hu Y., Wen Y., Wang X., Wei Y., Sheng G., Wang G. (2024). The impacts of forest therapy on the physical and mental health of college students: a review. Forests.

[4] Bayram N., Bilgel N. (2008). The prevalence and socio-demographic correlations of depression, anxiety and stress among a group of university students. Soc. Psychiatr. Psychiatr. Epidemiol..

[5] Keyes C.L.M., Eisenberg D., Perry G.S., Dube S.R., Kroenke K., Dhingra S.S. (2012). The relationship of level of positive mental health with current mental disorders in predicting suicidal behavior and academic impairment in college students. J. Am. Coll. Health.

[6] Chen L., Wang L., Qiu X.H., Yang X.X., Qiao Z.X., Yang Y.J., Liang Y. (2013). Depression among Chinese university students: prevalence and socio-demographic correlates. PLoS One.

[7] Regehr C., Glancy D., Pitts A. (2013). Interventions to reduce stress in university students: A review and meta-analysis. J. Affect. Disord..

[8] Garlow S.J., Rosenberg J., Moore J.D., Haas A.P., Koestner B., Hendin H., Nemeroff C.B. (2008). Depression, desperation, and suicidal ideation in college students: results from the American Foundation for Suicide Prevention College Screening Project at Emory University. Depress. Anxiety.

[9] Li H., Du Z., Shen S., Du W., Kang J., Gong D. (2023). An RCT-reticulated meta-analysis of six MBE therapies affecting college students' negative psychology. iScience.

[10] Vujcic M., Tomicevic-Dubljevic J., Grbic M., Lecic-Tosevski D., Vukovic O., Toskovic O. (2017). Nature based solution for improving mental health and well-being in urban areas. Environ. Res..

[11] Elsadek M., Liu B., Lian Z. (2019). Green façades: Their contribution to stress recovery and well-being in high-density cities. Urban For. Urban Green..

[12] Bruno A., Arnoldi I., Barzaghi B., Boffi M., Casiraghi M., Colombo B., Di Gennaro P., Epis S., Facciotti F., Ferrari N. (2024). The One Health approach in urban ecosystem rehabilitation: An evidence-based framework for designing sustainable cities. iScience.

[13] Kaplan S. (1995). The restorative benefits of nature: Toward an integrative framework. J. Environ. Psychol..

[14] Kaplan S., Berman M.G. (2010). Directed attention as a common resource for executive functioning and self-regulation. Perspect. Psychol. Sci..

[15] Stevenson M.P., Schilhab T., Bentsen P. (2018). Attention Restoration Theory II: A systematic review to clarify attention processes affected by exposure to natural environments. J. Toxicol. Environ. Health B Crit. Rev..

[16] Ohly H., White M.P., Wheeler B.W., Bethel A., Ukoumunne O.C., Nikolaou V., Garside R. (2016). Attention Restoration Theory: A systematic review of the attention restoration potential of exposure to natural environments. J. Toxicol. Environ. Health B Crit. Rev..

[17] Ulrich R.S., Simons R.F., Losito B.D., Fiorito E., Miles M.A., Zelson M. (1991). Stress recovery during exposure to natural and urban environments. J. Environ. Psychol..

[18] Hartig T., Staats H. (2006). The need for psychological restoration as a determinant of environmental preferences. J. Environ. Psychol..

[19] Chun M.H., Chang M.C., Lee S.J. (2017). The effects of forest therapy on depression and anxiety in patients with chronic stroke. Int. J. Neurosci..

[20] Berto R. (2014). The role of nature in coping with psycho-physiological stress: a literature review on restorativeness. Behav. Sci..

[21] Rothacher J., Mitesser O., Müller S., Scherer-Lorenzen M., Buřivalová Z., Müller J. (2025). Testing the soundscape response to silvicultural interventions in a controlled before-and-after experiment. Biol. Conserv..

[22] Zhang J., Zuo J., Hui T., Song S., Yang Z. (2025). Empirical study on the effect of single insect sounds on human perception based on pressure and engagement indicators. Sci. Rep..

[23] Ma H., Shu S. (2018). An experimental study: The restorative effect of soundscape elements in a simulated open-plan office. Acta Acustica united Acustica.

[24] Pijanowski B.C., Villanueva-Rivera L.J., Dumyahn S.L., Farina A., Krause B.L., Napoletano B.M., Gage S.H., Pieretti N. (2011). Soundscape ecology: the science of sound in the landscape. Bioscience.

[25] Bhandawat A., Jayaswall K. (2022). Biological relevance of sound in plants. Environ. Exp. Bot..

[26] Buxton R.T., Pearson A.L., Allou C., Fristrup K., Wittemyer G. (2021). A synthesis of health benefits of natural sounds and their distribution in national parks. Proc. Natl. Acad. Sci. USA.

[27] Doser J.W., Hannam K.M., Finley A.O. (2020). Characterizing functional relationships between anthropogenic and biological sounds: a western New York state soundscape case study. Landsc. Ecol..

[28] Conniff A., Craig T. (2016). A methodological approach to understanding the wellbeing and restorative benefits associated with greenspace. Urban For. Urban Green..

[29] Park S.H., Lee P.J., Jung T., Swenson A. (2020). Effects of the aural and visual experience on psycho-physiological recovery in urban and rural environments. Appl. Acoust..

[30] Peterson M.N., Larson L.R., Hipp A., Beall J.M., Lerose C., Desrochers H., Lauder S., Torres S., Tarr N.A., Stukes K. (2024). Birdwatching linked to increased psychological well-being on college campuses: A pilot-scale experimental study. J. Environ. Psychol..

[31] Ratcliffe E. (2021). Sound and soundscape in restorative natural environments: A narrative literature review. Front. Psychol..

[32] Guo Y., Jiang X., Zhang L., Zhang H., Jiang Z. (2022). Effects of sound source landscape in urban forest park on alleviating mental stress of visitors: Evidence from Huolu Mountain Forest Park, Guangzhou. Sustainability.

[33] Luo L., Zhang Q., Mao Y., Peng Y., Wang T., Xu J. (2023). A study on the soundscape preferences of the elderly in the urban Forest parks of underdeveloped cities in China. Forests.

[34] Wang R., Zhao J. (2019). A good sound in the right place: Exploring the effects of auditory-visual combinations on aesthetic preference. Urban For. Urban Green..

[35] Alvarsson J.J., Wiens S., Nilsson M.E. (2010). Stress recovery during exposure to nature sound and environmental noise. Int. J. Environ. Res. Publ. Health.

[36] Liu Y., Hu M., Zhao B. (2019). Audio-visual interactive evaluation of the forest landscape based on eye-tracking experiments. Urban For. Urban Green..

[37] Wang P., He Y., Yang W., Li N., Chen J. (2022). Effects of soundscapes on human physiology and psychology in Qianjiangyuan National Park System Pilot Area in China. Forests.

[38] Hong X.C., Cheng S., Liu J., Dang E., Wang J.B., Cheng Y. (2022). The physiological restorative role of soundscape in different forest structures. Forests.

[39] Erfanian M., Mitchell A., Aletta F., Kang J. (2021). Psychological well-being and demographic factors can mediate soundscape pleasantness and eventfulness: A large sample study. J. Environ. Psychol..

[40] Zhang Z., Chen Y., Qiao X., Zhang W., Meng H., Gao Y., Zhang T. (2022). The influence of forest landscape spaces on physical and mental restoration and preferences of young adults of different genders. Forests.

[41] Yu L., Kang J. (2010). Factors influencing the sound preference in urban open spaces. Appl. Acoust..

[42] Liu P., Liu M., Xia T., Wang Y., Wei H. (2021). Can urban Forest settings evoke positive emotion? Evidence on facial expressions and detection of driving factors. Sustainability.

[43] Fan L., Baharum M.R. (2024). The effect of exposure to natural sounds on stress reduction: a systematic review and meta-analysis. Stress.

[44] Liu Y., Hu M., Zhao B. (2020). Interactions between forest landscape elements and eye movement behavior under audio-visual integrated conditions. J. For. Res..

[45] Aspinall P., Mavros P., Coyne R., Roe J. (2015). The urban brain: analysing outdoor physical activity with mobile EEG. Br. J. Sports Med..

[46] Qi Y., Chen Q., Lin F., Liu Q., Zhang X., Guo J., Qiu L., Gao T. (2022). Comparative study on birdsong and its multi-sensory combinational effects on physio-psychological restoration. J. Environ. Psychol..

[47] Dale A.M., Liu A.K., Fischl B.R., Buckner R.L., Belliveau J.W., Lewine J.D., Halgren E. (2000). Dynamic statistical parametric mapping: combining fMRI and MEG for high-resolution imaging of cortical activity. Neuron.

[48] Yuan J.J., Wang Y., JU E.X., Li H. (2010). Gender differences in emotional processing and its neural mechanisms. Adv. Psychol. Sci..

[49] Shen H., He X., He J., Li D., Liang M., Xie X. (2024). Back to the village: assessing the effects of naturalness, landscape types, and landscape elements on the restorative potential of rural landscapes. Land.

[50] Jap B.T., Lal S., Fischer P., Bekiaris E. (2009). Using EEG spectral components to assess algorithms for detecting fatigue. Expert Syst. Appl..

[51] Yin M., Li K., Xu Z., Jiao R., Yang W. (2024). Exploring the impact of autumn color and bare tree landscapes in virtual environments on human well-being and therapeutic effects across different sensory modalities. PLoS One.

[52] Chen C., Li K., Wu Q., Wang H., Qian Z., Sudlow G. (2013). EEG-based detection and evaluation of fatigue caused by watching 3DTV. Displays.

[53] Cohen S., Kamarck T., Mermelstein R. (1983). A global measure of perceived stress. J. Health Soc. Behav..

[54] Zhang N., Liu C., Zhang M., Guan Y., Wang W., Liu Z., Gao W. (2025). Effects of traffic noise on the psychophysiological responses of college students: An EEG study. Build. Environ..

[55] Ratcliffe E., Gatersleben B., Sowden P.T. (2013). Bird sounds and their contributions to perceived attention restoration and stress recovery. J. Environ. Psychol..

[56] Nordh H., Hagerhall C.M., Holmqvist K. (2013). Tracking restorative components: patterns in eye movements as a consequence of a restorative rating task. Landsc. Res..

[57] Medvedev O., Shepherd D., Hautus M.J. (2015). The restorative potential of soundscapes: A physiological investigation. Appl. Acoust..

[58] Krzywicka P., Byrka K. (2017). Restorative qualities of and preference for natural and urban soundscapes. Front. Psychol..

[59] Liu C., Jing X., Shi J., Li J., Zhang Y., Gao W. (2024). Effects of natural sound on human stress recovery based on EEG techniques. J. Environ. Psychol..

[60] Gomez P., Danuser B. (2004). Affective and physiological responses to environmental noises and music. Int. J. Psychophysiol..

[61] Fisher A.J., Granger D.A., Newman M.G. (2010). Sympathetic arousal moderates self-reported physiological arousal symptoms at baseline and physiological flexibility in response to a stressor in generalized anxiety disorder. Biol. Psychol..

[62] Chen Y., Pan L., Lu F., Sun D., Liao C., Na M. (2025). Psychological Detachment in Chinese Higher Education: A Multitheoretical Model of Academic Stress, Cultural Pressure, and Coping Resources. Front. Psychol..

[63] Liang Q., Lin S., Wang L., Yang F., Yang Y. (2024). The impact of campus soundscape on enhancing student emotional well-being: A case study of fuzhou university. Buildings.

[64] Annerstedt M., Jönsson P., Wallergård M., Johansson G., Karlson B., Grahn P., Hansen Å.M., Währborg P. (2013). Inducing physiological stress recovery with sounds of nature in a virtual reality forest—Results from a pilot study. Physiol. Behav..

[65] Hwang S., Jebelli H., Choi B., Choi M., Lee S. (2018). Measuring workers’ emotional state during construction tasks using wearable EEG. J. Constr. Eng. Manage..

[66] Zhang Y., Zhang M., Fang Q. (2019). Scoping review of EEG studies in construction safety. Int. J. Environ. Res. Publ. Health.

[67] Zhang Y., Huang Y., Zheng M., Zhang H., Zhang Q., He T., Ye J. (2024). A study on the characteristics and influencing factors of soundscape perception in landscape spaces of urban greenways. Forests.

[68] Yang H., Zhang S. (2024). Impact of rural soundscape on environmental restoration: An empirical study based on the Taohuayuan Scenic Area in Changde, China. PLoS One.

[69] Fang X., Gao T., Hedblom M., Xu N., Xiang Y., Hu M., Chen Y., Qiu L. (2021). Soundscape perceptions and preferences for different groups of users in urban recreational forest parks. Forests.

[70] Simon-Dack S.L., Friesen C.K., Teder-Sälejärvi W.A. (2009). Sex differences in auditory processing in peripersonal space: an event-related potential study. Neuroreport.

[71] Svobodova K., Sklenicka P., Molnarova K., Salek M. (2012). Visual preferences for physical attributes of mining and post-mining landscapes with respect to the sociodemographic characteristics of respondents. Ecol. Eng..

[72] Guo M., Zhang J., Yang Z., Fan C., Zuo J., Hui T., Mao A., Qi J. (2024). An empirical study on the response of university students to viewing autumn secondary forest phytocommunities landscape via virtual reality in Northeast China. Ecol. Indic..

[73] Jin Y., Jin H., Kang J. (2020). Effects of sound types and sound levels on subjective environmental evaluations in different seasons. Build. Environ..

[74] Wu H., Chen Z., Wang G., Tang X. (2025). Does forest immersion benefit everyone? Investigating baseline stress and scene type as thresholds for neuropsychological restoration. Trees For. People.

[75] van Jaarsveld C.H.M., Fidler J.A., Steptoe A., Boniface D., Wardle J. (2009). Perceived stress and weight gain in adolescence: a longitudinal analysis. Obesity.

[76] Deng L., Rising H.H., Gu C., Bimal A. (2024). Effects of labyrinth-like path designs on mitigating stress response to traffic noise. Build. Environ..

[77] Forschack N., Oxner M., Müller M.M. (2025). The consequences of color chromaticity on electrophysiological measures of attentional deployment in visual search. iScience.

[78] Guo X., Jiang S.Y., Liu J., Chen Z., Hong X.C. (2024). Understanding the Role of Visitor Behavior in Soundscape Restorative Experiences in Urban Parks. Forests.

[79] Kang J., Zhang M. (2010). Semantic differential analysis of the soundscape in urban open public spaces. Build. Environ..

[80] Aletta F., Kang J., Axelsson Ö. (2016). Soundscape descriptors and a conceptual framework for developing predictive soundscape models. Landsc. Urban Plann..

[81] Payne S.R. (2013). The production of a perceived restorativeness soundscape scale. Appl. Acoust..

[82] Korpela K.M., Ylén M., Tyrväinen L., Silvennoinen H. (2008). Determinants of restorative experiences in everyday favorite places. Health Place.

[83] Davies W.J., Adams M.D., Bruce N.S., Cain R., Carlyle A., Cusack P., Hall D.A., Hume K.I., Irwin A., Jennings P. (2013). Perception of soundscapes: An interdisciplinary approach. Appl. Acoust..

[84] Frescura A., Lee P.J., Jeong J.H., Soeta Y. (2023). EEG alpha wave responses to sounds from neighbours in high-rise wood residential buildings. Build. Environ..

[85] Grassini S., Revonsuo A., Castellotti S., Petrizzo I., Benedetti V., Koivisto M. (2019). Processing of natural scenery is associated with lower attentional and cognitive load compared with urban ones. J. Environ. Psychol..

[86] Hamada M., Zaidan B.B., Zaidan A.A. (2018). A systematic review for human EEG brain signals based emotion classification, feature extraction, brain condition, group comparison. J. Med. Syst..

