# Supplementary material for: Effects of forest park’s biological sounds on the positive responses of stressed university student
Source: iScience. 2026 May 24;29(6):116073. doi: 10.1016/j.isci.2026.116073 (PMC13226232; doi:10.1016/j.isci.2026.116073)
Supplement: Data S1. Ethics approval document from the Medical Ethics Committee of Beihua University Hospital (ERC-2022-047) [file mmc1.pdf]

iScience, Volume *29*

Supplemental information

Effects of forest park's biological sounds on the positive responses of stressed university student

Tengfei Hui, Jie Zhang, Xuan Pei, Yang Zhi, Tong Song, Shiyu Song, Aiyun Zhu, Wei Gao, and Chunlan Zhang

The Ethical Approval for Application of Data from Human Subjects

This application is made and responsible for studies on observed data derived from all sources about human-being as subjects. This approval is signed as responsibility for a study involving Forestry College of Beihua University. This application is drafted by the initiator affiliated in Beihua University, and submitted to Ethic Broad Committee (EBC) of Affiliated Hospital of Beihua University will review and consent authorization if approved. Only when approval happened will this application be authorized to use data of human subjects in secured study according to requirements as follow:

Study theme: Effects of Biological sound in forest park on the psychophysiological responses of Stressed University Students

(i) Study methodology: Collect the EEG signals of college students who participated in the experiment when listening to biological sounds
(ii) Informed and consent: Participants sign informed consent before experiment
(iii) Data source and treatment: EEG data of collected will be used as source of data for statistical analysis.
(iv) Right and responsibility: initiator and sponsor of this statement have the right to use relevant data for human studies, which have been authorized and continuously supervised by EBC. If any terms in this file were violated, should the initiator and sponsor together be responsible for all possible results with irrefutable acceptance of irrelevance of any responsibilities from EBC.

The current approval would violate consents of study subjects and signature holder immediately when one of the followings aspects came true:

(i) The dataset by the authorization of this approval was found to be public or used by other institutions.
(ii) The privacy of any objectives involved in this study was revealed.
(iii) Results analyzed by the dataset were found to be tampered or misused for the other approaches rather than the current study.

To sign as a full awareness and an agreement to all above contents:

Initiator: Tengfei Hui

Signature: Tengfei Hui

To sign as a post-review approval to consent the authorization of all above contents:

EBC supervisors:

Fang Deng

Date: 10 August, 2024
